# A randomized controlled trial of physical activity, dietary habit, and distress management with the Leadership and Coaching for Health (LEACH) program for disease-free cancer survivors

**DOI:** 10.1186/s12885-017-3290-9

**Published:** 2017-05-02

**Authors:** Young Ho Yun, Young Ae Kim, Myung Kyung Lee, Jin Ah Sim, Byung-Ho Nam, Sohee Kim, Eun Sook Lee, Dong-Young Noh, Jae-Young Lim, Sung Kim, Si-Young Kim, Chi-Heum Cho, Kyung Hae Jung, Mison Chun, Soon Nam Lee, Kyong Hwa Park, Sohee Park

**Affiliations:** 1 Department of Biomedical Sciences, Seoul National University College of Medicine, Seoul, South Korea; 2 Cancer Research Institute, Seoul National University Hospital and College of Medicine, Seoul, South Korea; 3 Research Institute and Hospital, National Cancer Center, Goyang, South Korea; 4 College of Nursing, Kyungpook National University, Daegu, South Korea; 5 Cancer Biostatistics Branch, Research Institute for National Cancer Control and Evaluation, National Cancer Center, Goyang, South Korea; 6 Cancer Research Institute, Department of Surgery, Seoul National University College of Medicine, Seoul, South Korea; 7 Department of Rehabilitation Medicine, Seoul National University Bundang Hospital, Seoul National University College of Medicine, Bundang, South Korea; 8 Department of Surgery, Sungkyunkwan University School of Medicine, Seoul, South Korea; 9 Department of Internal Medicine, Kyung Hee University Hospital, Seoul, South Korea; 10 Department of Obstetrics and Gynecology, Keimyung University School of Medicine, Daegu, South Korea; 11 Department of Oncology, Asan Medical Center, University of Ulsan College of Medicine, Seoul, South Korea; 12 Department of Radiation Oncology, Ajou University School of Medicine, Suwon, South Korea; 13 Department of Internal Medicine, Ewha Womans University School of Medicine, Seoul, South Korea; 14 Division of Medical Oncology, Department of Internal Medicine, Korea University College of Medicine, Seoul, South Korea; 15 Department of Epidemiology and Health Promotion, Yonsei University Graduate School of Public Health, Seoul, South Korea

**Keywords:** Health partnership, Health coaching, Balanced diet, Regular exercise, Positive thinking

## Abstract

**Background:**

We aimed to evaluate the potential benefits of the Leadership and Coaching for Health (LEACH) program on physical activity (PA), dietary habits, and distress management in cancer survivors.

**Methods:**

We randomly assigned 248 cancer survivors with an allocation ratio of two-to-one to the LEACH program (LP) group, coached by long-term survivors, or the usual care (UC) group. At baseline, 3, 6, and 12 months, we used PA scores, the intake of vegetables and fruits (VF), and the Post Traumatic Growth Inventory (PTGI) as primary outcomes and, for secondary outcomes, the Ten Rules for Highly Effective Health Behavior adhered to and quality of life (QOL), the Hospital Anxiety and Depression Scale (HADS), and the European Organization for Research and Treatment of Cancer Quality of Life Questionnaire (EORTC QLQ-C30).

**Results:**

For primary outcomes, the two groups did not significantly differ in PA scores or VF intake but differed marginally in PTGI. For secondary outcomes, the LP group showed a significantly greater improvement in the HADS anxiety score, the social functioning score, and the appetite loss and financial difficulties scores of the EORTC QLQ-C30 scales from baseline to 3 months. From baseline to 12 months, the LP group showed a significantly greater decrease in the EORTC QLQ-C30 fatigue score and a significantly greater increase in the number of the Ten Rules for Highly Effective Health Behavior.

**Conclusion:**

Our findings indicate that the LEACH program, coached by long-term survivors, can provide effective management of the QOL of cancer survivors but not of their PA or dietary habits.

**Trial registration:**

Clinical trial information can be found for the following: NCT01527409 (the date when the trial was registered: February 2012).

**Electronic supplementary material:**

The online version of this article (doi:10.1186/s12885-017-3290-9) contains supplementary material, which is available to authorized users.

## Background

As a result of the substantial progress made in the early detection of cancer and new treatment technologies, the population of cancer survivors is increasing [1, 2]. Unfortunately, however, many cancer survivors develop poor health behaviors, such as physical inactivity, and exhibit overweight and psychological distress [3–5], and many develop recurrent or secondary primary cancers [6–9] during the transition from intensive treatment to survivorship.

Cancer can now be viewed as a chronic illness subject to management and long-term surveillance [10]. The US Institute of Medicine’s (IOM) new paradigm for the Chronic Care Model (CCM) of survivorship care planning (SCP) requires an ongoing collaborative partnership between patients and providers [10, 11]. These partnerships empower cancer patients by enabling them to manage their health crisis and quality of life (QOL) through self-management interventions [12, 13]. As in the proactive leadership trend in organizational management, self-leadership could empower patients to maintain healthy habits and grow positively in a CCM [3]. Since self-leadership, healthy behaviors, and post-traumatic growth factors are associated with health-related quality of life (HRQOL) in cancer survivors [3], we are developing a novel health management program based on self-leadership that is designed to empower patients to proactively improve their health.

Another model for careful and proactive health management tailored to each patient’s health status and preference is health coaching, a hospital-based program that provides regular coaching sessions to patients by phone [14, 15]. The novel, trans-theoretical model (TTM)-based health management program we designed to empower cancer survivors to take care of themselves is called “Leadership and Coaching for Health” (LEACH).

Here we describe a 12-month randomized control trial that evaluated the benefits of LEACH on physical activity, dietary habits, and distress management compared with the benefits of routine care (standardized health education materials and a workshop) in a large sample of patients at 10 teaching hospitals, each with a different health partner and health master coach. Our hypothesis was that patients using LEACH would show increased physical activity, adopt a better diet, and attain greater positive growth than patients who received routine care.

## Methods

### Study design

This was a randomized controlled trial that evaluated the efficacy of a stage-tailored intervention based on the LEACH program from April 2012 through August 2013, using usual care as a control. The LEACH program consists of comprehensive, multifaceted core strategies from the TTM of health behavior change, the leadership model of “Seven Habits of Highly Effective People” [16], and a Coaching Model [17]. The intervention includes 1) a TTM-based health education booklet and workbook for cancer survivors, 2) a workshop for empowerment of patients’ leadership skills, and 3) TTM-based telephone coaching with a health coaching manual (repeated assessment of stage of change, and planning how to achieve target health levels in accordance with their preferences and abilities) (Additional file 1: Table S1). The LEACH program covers physical activity, diet, and distress management.

### Eligibility criteria

We used cancer registries from 10 South Korean teaching hospitals, each with a different health partner and health master coach. Cancer survivors who completed primary cancer treatment (in situ, localized, or regional with a favorable prognosis) within the last 24 months for breast, stomach, colon (other than rectal), and lung cancer within 18 months of completion of primary treatment were identified. To be included in the study, a patient had to 1) be ≥20 years old, 2) have a platelet count ≥100,000/mm^3^, 3) have a serum hemoglobin ≥10 g/dl, and 4) have not already met two or more behavioral goals aimed for in the study (i.e., i) energy expenditure achieved by at least moderate exercise for at least 150 min/week; ii) intake of ≥5 servings of fruit and vegetables per day; iii) a total score > 72 points in the Post Traumatic Growth Inventory). Patients were excluded from the study if they 1) were currently receiving cancer treatment, 2) had a progressive malignant disease or a recurrent, metastasized, or additional primary cancer, 3) had a condition that might compromise adherence to an unsupervised exercise program (e.g., uncontrolled congestive heart failure or angina, recent myocardial infarction, breathing difficulties requiring oxygen use or hospitalization, unable to walk without a walker or wheelchair, or were planning to receive hip or knee replacement surgery), 4) had a condition that could interfere with ingestion of a diet high in vegetables and fruit (e.g., kidney failure or need for chronic warfarin, 5) a serious psychological disorder (e.g., bipolar disease, schizophrenia, or an eating disorder), 6) had an infection (body temperature ≥ 37.2 °C or WBC ≥11,000 mm^3^), 7) had visual or motor dysfunction, or 8) were pregnant.

### Participant recruitment

Permission to contact patients was obtained from the patients’ physician. Recruitment was either by a mailed letter of invitation or by direct approach by a research staff member in an outpatient department in a study hospital. The letters of invitation, which were stamped with an official approval seal from the Institutional Review Board, included an explanation of the LEACH study, a LEACH study promotional leaflet, a preaddressed, postage-paid return envelope, and a brief instrument that screened for ineligibility factors. After the prescreening, an oncologist and a research staff member in each study hospital confirmed that patients met the eligibility criteria by reviewing medical records and by blood tests. Research staff then related the details of the study to participants who met the eligibility criteria and who provided written informed consent. This study was approved by the Institutional Review Boards of each hospital.

### Random assignment

With the aid of a computerized random number generator (SAS 9.1.3, Proc plan), we randomly assigned eligible participants, two-to-one, to the intervention or the usual care group. To minimize the effects of potentially confounding variables on outcomes, we performed block randomization with 8 strata defined by type of cancer (breast, stomach, colon, or lung) and number of behavior goals practiced at the study entry (0 or 1 out of 3 defined possible behaviors).

### Training programs for health master coach and health partner

For the LEACH study, we developed two training programs: the “Health Master Coach Program” for professionals and the “Health Partner Program” for long-term cancer survivors. “Health Partners” were trained by the “Health Partner Program” and are mentored and supervised by a Health Master Coach. Health Master Coaches were trained by the Health Master Coach Program, which consists of three components, such as education on health management in survivorship care planning (i.e., regular exercise, balanced diet, distress management, regular screening, no smoking and drinking, and management of chronic fatigue), leadership, coaching, and facilitator training. The education on the teaching and learning methods was a 72-h group session in parallel with actual practice. The health partners had been trained by the “Health Partner Program,” which is 3-month program consisting of health behavior management (8 h), leadership (16 h), and actual health coaching practice on prior learning via eight sessions using a multilateral telephone system (24 h).

### Study conditions

#### Intervention (LEACH)

The LEACH program is based on 3 concepts—health education, leadership, and coaching, and it is managed through the interaction of a health master coach, health partners, and cancer patients. Health partners were long-term cancer survivors who formed partnerships with cancer patients and helped them achieve the target levels set for their health behaviors. Health master coaches were health professionals who mentored and supervised health partners.

First, patients were given a 1-h health education workshop (physical activity, dietary habits, and distress management) and a 3-h leadership workshop (Seven Habits of Highly Effective People with Cancer). Next, the Intervention group was also offered individual coaching by telephone for a 24-week period. A total of 16 sessions of tele-coaching were conducted: 30 min per week for 12 sessions, 30 min per 2 weeks for 2 sessions, and 30 min per month for 2 sessions were offered for the intervention group. Throughout the LEACH program, participants in the intervention group were provided individual coaching by telephone to practice patient health behaviors (such as regular exercise, balanced diet, and positive thinking) that have been reported to help in self-management. Based on the baseline health status assessment, health partners kept written records of their coaching, and master coaches gave feedback by reviewing those records. The principle investigator supervised these processes. The aim of the intervention was to achieve success in more than two health behaviors among three primary outcomes (physical activity ≧12.5 metabolic equivalents of task (METs) hours per week, daily intake of fruit and vegetables ≧5 dishes per day, and total PTGI ≧72). The secondary outcomes were to improve QOL and leadership of cancer survivors.

### Intervention materials

#### Health education materials

Health education materials based on the TTM of health behavior change included information about 3 intervention areas—physical activity, dietary habits, and distress management. We made the material easy for health partners to understand so that they, in turn, could make it easy for patients to understand.

#### Health leadership-coaching workbook

Typically, patient health education does not involve interactions. We changed this by developing a leadership-coaching workbook that patients could work with to target their goal, set their action plan, and practice health leadership skills. The health education material in the workbook was based on a TTM model, self-leadership, and coaching strategy. The workbook was provided to health partners and patients.

#### Health coaching manual

Since health partners were not coaching professionals, we developed a coaching manual that they could use to guide patients on how to achieve the target levels for their health in accordance with their preferences and abilities using a TTM model, self-leadership, and coaching strategy.

### Control material

The control group was encouraged to continue their usual care and was given a health education booklet on physical activity, dietary habits, and distress management that was not based on the core strategies from the TTM of health behavior change, as well as a 4-h health education lecture on physical activity, dietary habits, distress management, and screening for a 2nd cancer.

### Quality assurance

Quality assurance covered study personnel, Health Partners [18], Health Master Coach training programs [18], experts’ supervision of the LEACH interventions, and the quality assurance committees. All research staff involved in screening and recruiting participants passed their local institution certification requirements for the ethical conduct of research (The Collaborative Institutional Training Initiatives).

### Primary outcome

The patients were evaluated at 0, 3, 6, and 12 months. However, due to the lack of participants in the 6-month period, we did not include the 6-month follow-up results in the statistical analyses for this study. The primary outcomes were improvements in physical activity, diet, and post-traumatic growth. Physical activity was measured in METs (kcal/kg/week) using survey responses about the time, length, and intensity of physical activity [19] following the ACSM’s guidelines for exercise testing and prescription [20]. Diet was evaluated with validated questions about daily intake of vegetables and fruits, and dietary pattern was checked with a questionnaire based on the “Rules for National Cancer Prevention: Dietary Practice Guideline,” which contains 10 questions exploring nutrition balance and dietary habits, such as eating speed and frequency [21, 22]. To evaluate diet, the survey questionnaire was modified based on the Korean National Health and Nutrition Examination Survey data [23]. Posttraumatic growth was measured with the Posttraumatic Growth Inventory (PTGI), a 21-item scale that assesses positive outcomes from persons who experienced traumatic events [24]. Each item was scaled on a 6-point Likert score from 0 to 5.

### Secondary outcomes

The secondary outcomes were improvement in leadership, HRQOL, satisfaction with life, depression and anxiety, distress in response to a specific traumatic event, perceived social support, and number of the Ten Rules for Highly Effective Health Behavior adhered to. All of the secondary outcome questionnaires were validated in a Korean version with cancer survivors [3, 25, 26].

### Cancer survivors’ leadership

We measured the 7 habits of highly effective people with cancer using the Seven Habit Profile (7HP) [16]. Each question was scored on a 6-point Likert scale, with the sum of the 3 questions covering one subscale, therefore, the total score of each domain was 18. The total of 27 questions consists of 9 subscales, higher scores representing the closer alignment with leadership criteria.

### Health related quality of life

HRQOL was assessed using the 30-item European Organization for Research and Treatment of Cancer Quality of Life Questionnaire C30 (EORTC QLQ-C30) based on a 4-point Likert scale [26, 27]. Global life satisfaction was assessed using Diener’s Satisfaction with Life Scale (SWLS), which is scored from 1 to 7 so that the possible range is from 5 to 35; higher scores indicate higher satisfaction [28]. Psychological distress was assessed using the Hospital Anxiety and Depression Scale (HADS) [29]; total scores range from 0 to 21 for each of the anxiety and depression subscales. Self-reported current subjective cancer-induced distress in response to a specific traumatic event was rated using the Impact of Events Scale-Revised (IES-R) [25]. The 22-item scale is composed of 3 subscales representative of the major symptom clusters of post-traumatic stress and this questionnaire is scored with 5-point Likert scales, which comprise 0 (not at all), 1 (a little bit), 2 (moderately), 3 (quite a bit), and 4 (extremely). Perceived social support was assessed using the 20-item Medical Outcomes Study Social Support Survey (MOS-SSS) [30]. To obtain a score for overall support, the average of all 19 item scores are calculated and then can be transformed to a 0–100 scale; however, one item rates the number of close friends or relatives.

Patients were also asked to rate how they applied the following Ten Rules for Highly Effective Health Behavior [3] (i.e., positive thinking, regular exercise, balanced diet, etc.) to improve QOL. Health behavior stages (pre-contemplation, contemplation, preparation, action, and maintenance) were based on the TTM [31]. Behavior stages range from 1 (pre-contemplation) to 5 (maintenance stage) for each item [31].

## Statistical approach

Anticipating a 20% dropout rate, we set the recruitment goal to 248 participants based upon the following assumptions: 1) a 2-sided Type I error of 0.05, 2) a 5% attainment of goal behavior in the usual care group (estimated Hawthorne effect), a 15–34% [32] attainment in the LEACH group, and a power of 78–89% to detect a between-arm difference. To achieve statistical power of 80% and an effect size of 0.3 by a two-sided *t* test, a 0.05 α level was used.

We explored intervention effects using an intent-to-treat analysis (ITT) that compared data from the original randomized groups regardless of group assignment. We used frequencies, means, SDs, and ranges to describe group characteristics, and the *t*-test (for continuous variables) and Chi-square test (for categorical variables) to evaluate homogeneity of the baseline characteristics between the two groups. We also analyzed each group’s success rate for combined primary outcomes. We calculated the rates of those who succeeded in performing more than two behaviors among three combined outcomes (physical activity ≧12.5 MET hours per week, daily intake of fruit and vegetables ≧5 dishes per day, and total PTGI ≧72) and compared these results between the UC and LP groups. Finally, analysis of covariance (ANCOVA) adjusting for baseline scores was conducted to compare between-group differences at each time point (3 and 12 months). The factors we explored were level of physical activity, body composition, diet quality, post-traumatic growth, self-leadership, satisfaction with life, HRQOL, anxiety and depression, and disease impact. For all statistical analyses, we included data for participants who completed the baseline questionnaire regardless of follow-up loss. All analyses were done with STATA version 13 (StataCorp LP, TX, USA) and SAS statistical package version 9.3 (SAS Institute Inc., Cary, NC), and all *p* values were two-sided.

## Results

### Participants

**Fig. 1:**
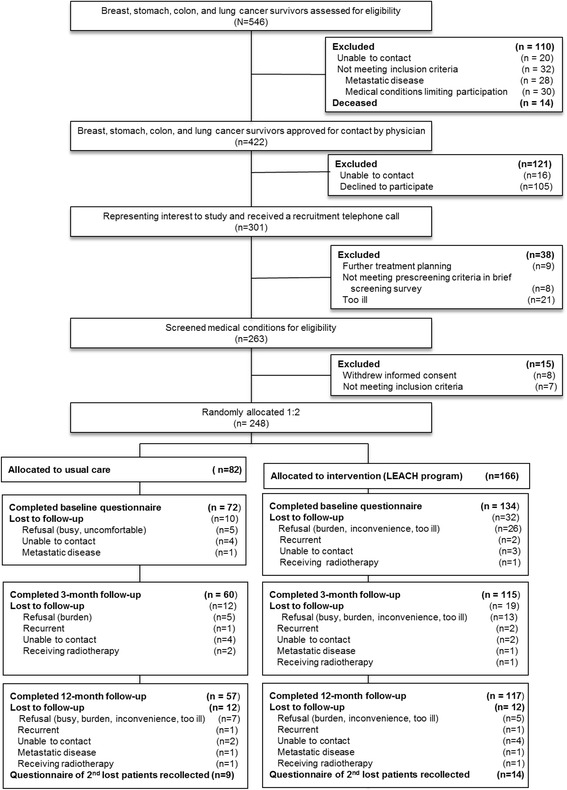
Flow diagram of participants: recruitment and eligibility screening, randomization, follow-up, and analyses

Of the 546 eligible patients, 298 were excluded for various reasons, leaving 248 (45.4%) for randomization into the study (Fig. 1). In the LP group, 115 (69.3%) participants completed the 12-month course at 3 months and 117 (70.5%) at 6–12 months. In the UC group, 60 (73.2%) participants completed the course at 3 months and 57 (71.3%) at 12 months.

### Baseline characteristics of participants

Table 1 summarizes the baseline characteristics of all participants. The scores of the two groups did not differ significantly for primary (PA, diet, and post-traumatic positive growth) or secondary (HADS, EORTC QLQ-C30, 7 Habit Profile, and MOS-SS) outcome measures (Table 1). The baseline questionnaire was completed by 72 (82.8%) participants in the UC group and 134 (80.72%) in the LP group.

**Table 1:** Baseline characteristics of the patients

Characteristic	Control group	Intervention group	All participants	*P*-value
	(*N* = 72)	(*N* = 134)	(*N* = 206)	
No. Coaching attendees		10.63 (6.29)		
Age, years	51.04 (7.55)	50.52 (10.21)	50.68 (9.43)	
Sex - no.(%)				
Male	18 (25.00)	24 (17.91)	42 (20.39)	0.229
Female	54 (75.00)	110 (82.09)	164 (79.61)	
Marital status- no.(%) (missing = 2)				
Married	63 (87.50)	106 (80.30)	169 (82.84)	0.193
Widowed/Divorced/separated/single	9 (12.50)	26 (19.70)	35 (17.16)	
Education - no.(%) (missing = 2)				
High-school graduate or less	39 (54.17)	66 (50.00)	105 (51.47)	0.569
College graduate	33 (45.83)	66 (50.00)	99 (48.53)	
Religion - no.(%) (missing = 4)				
No	16 (22.86)	47 (41.17)	64 (31.19)	0.063
Yes	54 (77.14)	85 (64.39)	139 (68.81)	
Household income –no.(%) (missing = 4)				
< 300million won	25 (34.72)	49 (37.69)	49 (47.62)	0.675
≥ 300million won	47 (65.28)	81 (82.38)	128 (63.37)	
Cancer stage (missing = 7)				
0	2 (2.94)	3 (2.29)	5 (2.51)	0.476
I	31 (45.59)	69 (52.67)	100 (52.67)	
II	28 (41.19)	38 (29.01)	66 (33.17)	
III	4 (5.88)	16 (12.21)	20 (10.05)	
IV	1 (1.47)	1 (0.76)	2 (1.01)	
Other (5,6)	2 (2.94)	4 (3.05)	6 (3.02)	
Type of cancer				
Stomach	17 (23.94)	34 (25.76)	51 (25.12)	
Lung	3 (2.80)	5 (5.20)	5 (3.79)	
Breast	42 (59.15)	81 (79,98)	123 (60.59)	
Colorectal	5 (7.04)	6 (4.55)	11 (5.42)	
Gynecologic	4 (5.63)	5 (3.79)	9 (4.43)	
Other	0 (0)	1 (0.76)	1 (0.49)	
Type of treatment (missing = 10)				
Surgery	68 (98.55)	127 (100.00)	195 (99.49)	0.174
Radiotherapy	39 (56.52)	61 (64.80)	100 (51.02)	0.256
Chemotherapy	43 (62.32)	76 (59.84)	119 (60.71)	0.735
Hormonal therapy	20 (50.0)	31 (40.26)	51 (43.59)	0.314
Weight, kg (missing = 1)	56.31 (8.36)	57.53 (8.72)	57.10 (8.59)	0.330
BMI (missing = 1)	21.70 (2.67)	22.23 (3.00)	22.05 (2.89)	0.213
Hemoglobin (missing = 14)	13.05 (1.25)	13.06 (1.22)	13.05 (1.23)	0.955
Total Cholesterol-mg/dl (missing = 21)	173.72 (28.62)	181.9 (34.60)	178.96 (11.44)	0.103
Systolic blood pressure, mm Hg (missing = 87)	116.33–72.94	112.9–72.93	117.52–72.94	0.462/0.999
Baseline				
Vegetable intake 5 plates/day, no. (%)				
Yes	22 (30.56)	45 (33.58)	67 (32.52)	0.659
No	50 (69.44)	89 (66.42)	139 (67.48)	
Total-MET	27.53 (19.82)	26.67 (27.50)	26.97 (25.04)	0.799
Posttraumatic Growth Inventory				
Relating to others	20.83 (6.17)	21.40 (6.64)	21.21 6.47)	0.568
New possibilities	14.36 (5.10)	14.61 (4.70)	14.52 (4.83)	0.723
Personal strength	11.28 (4.37)	11.90 (4.01)	11.68 (4.14)	0.308
Spiritual change	5.07 (3.09)	4.72 (4.20)	4.84 (3.08)	0.444
Appreciation for life	10.21 (3.18)	10.46 (2.92)	10.37 (3.01)	0.576
Total	61.78 (18.91)	63.09 (18.51)	62.63 (18.61)	0.631

Unless otherwise indicated, values = mean (SD)

*MET* metabolic equivalent task, *BMI* body mass index

### Effect of health partnership program

Table 2 shows each LP and UP group’s change of success rates in more than 2 of 3 primary outcomes (physical activity, dietary habits, and post-traumatic positive growth) from baseline to 3 and 12 months. The Chi-square test for each time point shows that the two groups did not differ significantly, however, in two or more health behavior goals. Table 3 shows the changes from baseline to 3 and 12 months in the two groups for all measures. For primary outcome scores, the two groups did not significantly differ in intake of vegetables and fruit (servings/day) (*p* = 0.819 for 3 months, and *p* = 0.413 for 12 months) and MET/h/day (*p* = 0.54 for 3 months, and *p* = 0.975 for 12 months), but differed marginally at 12 months in post-traumatic positive growth (*p* = 0.065). For secondary outcomes, the LP group showed a significantly greater decrease in the HADS anxiety score (*p* = 0.025), a significantly greater increase in the social functioning score of the EORTC QLQ-C30 (*p* = 0.018), and a significantly greater decrease in the appetite loss (*p* = 0.048) and financial difficulties scores (*p* = 0.036) of the EORTC QLQ-C30 from baseline to 3 months. From baseline to 12 months, the LP group, relative to the UC group, showed a significantly greater decrease in the EORTC QLQ-C30 fatigue score (*p* = 0.065) and a significantly greater increase in number of 10 Rules for Highly Effective Health Behavior adhered to (*p* = 0.015). Differences in IES-R score between the UC and LP groups were marginally significant from baseline to 12 months (*p* = 0.068).

**Table 2:** Primary outcomes over time

Time point	Intervention group		Control group		*P*-value^†^
	Total no.	Success no. (%)	Total no.	Success no. (%)	
Baseline	134	47 (35.1)	72	25 (34.7)	0.960
3 months	100	44 (44.0)	55	20 (36.4)	0.356
12 months	92	42 (45.7)	50	16 (32.0)	0.114

Each time point includes three primary outcomes (MET, PTGI, Vegetable intake 5 plates/day), with two or more defining success

^†^Chi-square test

**Table 3:** Effect of health partnership program

		Unadjusted estimates, mean (SD)		Adjusted analysis for intervention vs usual care^a^		
		Intervention group	Control group	Intervention group	Control group	*P* value^1)^
MET/h/day	Baseline	26.7 (27.5)	27.5 (19.8)			
	3 months	27.1 (25.4)	24.3 (24.6)	26.2 (2.2)	23.9 (3.0)	0.540
	12 months	23.5 (23.6)	22.9 (24.6)	22.9 (2.3)	22.8 (3.3)	0.975
PTGI-Total	Baseline	63.1 (18.5)	61.8 (18.9)			
	3 months	63.6 (19.3)	61.2 (18.6)	62.7 (1.3)	62.1 (1.7)	0.791
	12 months	66.6 (19.3)	60.2 (19.1)	66.3 (1.6)	61.2 (2.2)	0.065*
Vegetable intake n(%), ≥5 serves/day	Baseline	45 (33.6)	22 (30.6)			
	3 months	52 (42.6)	27 (40.9)	33.6 (4.9)	31.0 (6.3)	0.819
	12 months	41 (36.9)	26 (42.6)	31.0 (5.4)	37.3 (6.7)	0.413
Leadership	Baseline	121.3 (21.2)	120.1 (20.8)			
	3 months	125.0 (21.7)	121.6 (20.2)	123.1 (1.3)	121.8 (1.9)	0.552
	12 months	127.7 (22.1)	122.8 (20.6)	125.4 (1.6)	123.3 (2.2)	0.433
HADS						
Anxiety	Baseline	5.7 (3.4)	5.9 (3.1)			
	3 months	5.0 (3.0)	6.1 (3.1)	5.2 (0.2)	6.0 (0.3)	0.025**
	12 months	5.1 (3.0)	5.8 (2.9)	5.2 (0.3)	5.7 (0.4)	0.228
Depression	Baseline	6.4 (3.5)	6.1 (3.1)			
	3 months	5.5 (3.3)	5.4 (2.8)	5.6 (0.2)	5.6 (0.3)	0.986
	12 months	5.4 (3.4)	5.6 (3.1)	5.3 (0.3)	5.7 (0.4)	0.428
EORTC QLQ-C30						
Functional scales						
Global health status	Baseline	64.5 (19.9)	63.4 (18.7)			
	3 months	67.7 (18.7)	65.7 (17.5)	67.0 (1.6)	66.0 (2.3)	0.705
	12 months	70.1 (17.1)	65.3 (17.9)	69.0 (1.6)	66.0 (2.2)	0.269
Physical functioning	Baseline	78.6 (13.5)	77.9 (11.1)			
	3 months	80.0 (12.1)	78.4 (12.0)	79.4 (0.9)	79.3 (1.3)	0.942
	12 months	82.9 (13.1)	78.2 (12.4)	81.9 (1.2)	78.7 (1.6)	0.123
Role functioning	Baseline	79.4 (21.4)	77.9 (19.8)			
	3 months	80.9 (18.1)	77.3 (18.4)	80.3 (1.5)	78.5 (2.2)	0.497
	12 months	82.7 (19.8)	79.9 (18.9)	80.9 (1.8)	81.1 (2.4)	0.958
Emotional functioning	Baseline	76.8 (19.4)	73.0 (23.0)			
	3 months	78.0 (19.1)	74.5 (16.5)	76.7 (1.5)	75.3 (2.2)	0.602
	12 months	78.0 (19.9)	75.9 (18.3)	76.2 (1.9)	77.7 (2.4)	0.625
Cognitive functioning	Baseline	76.7 (19.9)	72.6 (20.9)			
	3 months	80.1 (17.2)	72.5 (20.2)	77.6 (1.4)	75.1 (2.4)	0.322
	12 months	78.1 (14.9)	76.5 (19.2)	76.8 (1.6)	78.4 (2.1)	0.552
Social functioning	Baseline	75.8 (26.8)	73.1 (23.4)			
	3 months	85.4 (19.3)	76.3 (20.2)	84.8 (1.8)	77.4 (2.5)	0.018
	12 months	85.3 (19.5)	78.2 (22.4)	84.8 (2.2)	79.0 (2.9)	0.123
Symptom scales						
Fatigue	Baseline	38.6 (20.9)	40.8 (21.6)			
	3 months	33.5 (17.8)	38.1 (17.7)	34.3 (1.5)	37.6 (2.2)	0.214
	12 months	33.8 (16.1)	42.4 (21.4)	34.8 (1.6)	41.9 (2.1)	0.010**
Nausea/vomiting	Baseline	4.6 (10.0)	4.2 (10.6)			
	3 months	5.7 (10.6)	5.3 (9.8)	5.6 (0.9)	6.2 (1.3)	0.733
	12 months	6.4 (14.5)	7.8 (15.3)	6.5 (1.6)	7.7 (2.1)	0.660
Pain	Baseline	15.4 (19.2)	21.4 (19.0)			
	3 months	11.9 (16.0)	19.6 (19.6)	13.6 (1.5)	17.4 (2.1)	0.146
	12 months	13.1 (17.6)	19.7 (21.4)	15.5 (1.8)	16.2 (2.3)	0.810
Dyspnea	Baseline	11.9 (19.9)	19.4 (21.0)			
	3 months	8.2 (15.9)	13.7 (16.6)	10.3 (1.3)	11.3 (1.9)	0.668
	12 months	10.6 (19.5)	13.6 (17.9)	12.1 (2.0)	11.3 (2.6)	0.797
Insomnia	Baseline	28.8 (30.0)	30.3 (28.9)			
	3 months	24.1 (24.50	26.7 (26.9)	25.0 (2.1)	25.7 (3.1)	0.850
	12 months	26.2 (27.9)	32.0 (27.2)	27.6 (2.5)	29.1 (3.4)	0.732
Appetite loss	Baseline	12.8 (20.9)	15.4 (24.8)			
	3 months	10.7 (17.6)	17.3 (23.6)	11.3 (1.8)	17.7 (2.6)	0.048
	12 months	11.6 (17.6)	13.6 (20.3)	12.1 (2.0)	13.7 (2.6)	0.631
Constipation	Baseline	16.9 (26.7)	18.9 (24.1)			
	3 months	14.7 (23.3)	16.3 (21.6)	16.5 (1.8)	16.0 (2.7)	0.882
	12 months	12.6 (18.1)	17.7 (20.5)	19.5 (2.2)	16.5 (2.9)	0.414
Diarrhea	Baseline	13.8 (20.7)	11.4 (18.8)			
	3 months	12.6 (18.1)	14.0 (17.9)	19.8 (4.1)	10.0 (5.4)	0.151
	12 months	20.5 (45.6)	9.5 (20.4)	11.9 (1.5)	15.3 (2.2)	0.211
Financial Difficulties	Baseline	20.3 (28.6)	23.4 (26.0)			
	3 months	15.5 (25.5)	26.8 (37.1)	17.4 (2.6)	27.0 (3.7)	0.036 **
	12 months	18.9 (26.6)	20.4 (27.9)	18.9 (2.5)	19.3 (3.3)	0.920
The MOS-SSS	Baseline	65.6 (20.9)	65.6 (20.6)			
	3 months	66.3 (21.1)	67.9 (19.3)	66.9 (1.4)	65.7 (2.0)	0.621
	12 months	66.6 (21.1)	68.0 (19.7)	67.1 (1.7)	65.3 (2.4)	0.535
Health Behavior	Baseline	4.9 (3.1)	5.2 (2.7)			
	3 months	6.4 (2.7)	5.5 (3.0)	6.9 (0.3)	6.2 (0.4)	0.147
	12 months	7.0 (2.8)	6.3 (2.7)	6.3 (0.2)	5.3 (0.3)	0.015**
IES-R	Baseline	2.3 (0.8)	2.5 (0.7)			
	3 months	2.2 (0.7)	2.2 (0.7)	2.3 (0.1)	2.2 (0.1)	0.759
	12 months	2.1 (0.7)	2.3 (0.8)	2.1 (0.1)	2.3 (0.1)	0.068 *

^a^Adjusted baseline value with a statistical power of 80% and an effect size of 0.3 by a two-sided *t* test at the 0.05 α level was used

*p < 0.10 with bold

**p < 0.05 with bold

^1)^ANCOVA

*MET* Metabolic Equivalent Task, *PTGI* Post-traumatic Growth Inventory, *HADS* Hospital and Anxiety Scale, *EROTC QLQ-C30* European Organization for Research and Treatment of Cancer Quality of Life Questionnaire C30, *MOS-SSS* Medical Outcomes Study Social Support Survey, *IES-R* Impact of Event Scale--Revised

Otherwise, other secondary outcomes such as depression (*p* = 0.9.86 for 3 months, and *p* = 0.428 for 12 months), QOL functioning (i.e., physical function, role function, emotional function, and cognitive function) and several symptom scales did not show statistically significant changes.

## Discussion

Despite the fact that cancer survivors are in a “teachable moment” at a time when they are highly motivated to change behaviors so as to improve their health [33], many practice poor health behaviors [34–37]. Therefore, in this program, we primarily targeted 3 intervention areas—physical activity, dietary habits, and post-traumatic positive growth—as well as secondary outcomes for health related quality of life and leadership improvement through health coaching. To our knowledge, this is the first health coaching program provided by long-term cancer survivors.

In this randomized controlled trial (RCT) of the Health Partnership program, the health partners’ tele-coaching significantly improved several areas of HRQOL but failed to change primary health behavior outcomes compared with routine care. There were no significant improvements for the 3 primary targeted intervention areas such as physical activity, dietary habits, and post-traumatic positive growth. The effect was evident in several sub-scales of three well-validated HRQOL assessment measures (EORTC QLQ-C30, HADS, and IES-R) as secondary outcomes. The observed benefits showed clinically significant improvements in fatigue, social functioning, anorexia, financial difficulties (EORTC QLQ-C30), anxiety (HADS), IES-R score, and health behavior activation numbers among long-term cancer survivors.

Although limited evidence has suggested that behavioral interventions for cancer survivors based on the TTM and cognitive behavioral therapy (CBT) can improve health outcomes, doubts remain about behavioral interventions to improve multiple behaviors simultaneously along with long-term health outcomes [38]. In RCTs based on TTM and CBT, our research team has showed that target goals could be improved by simultaneous stage-matched exercise and diet intervention [39], Health Navigation for cancer-related fatigue [40], and decision aids to help family caregivers discuss terminal disease status [41].

There are several possible explanations for our study findings. First, this study did not support our hypothesis that patients in the LEACH group would attain greater physical activity, intake of vegetables and fruit (servings/day), and post-traumatic positive growth than patients in the routine care group. In contrast to earlier RCTs of behavioral interventions for cancer survivors based on TTM, CBT, and health coaching, the LEACH program showed improvement only in secondary outcomes, such as anxiety, social functioning, anorexia, fatigue, financial difficulties, and the number of 10 Rules for Highly Effective Health Behavior adhered to [13, 42, 43].

We are particularly discouraged by the observation that the intervention and control groups did not differ in primary outcomes during the 12-month follow-up period. This may have been due to lower intervention intensity or low quality of coaching in the intervention arm. Our health coaching program intervention by long-term cancer survivors trained by the Health Partner Program may have important methodological limitations, including inadequate training of the health coach. We have tried to develop this program to provide a new paradigm of partnership between long-term cancer survivors and medical professionals to enable patients to manage their health crises and HRQOL across the cancer-care continuum. Specifically, our new model would enable long-term survivors to form partnerships between patients and physicians to make full use of their experience and wisdom gained during the “War with Cancer”; however, this study did not provide evidence of the effectiveness of the LEACH program.

Nonetheless, this study showed that Health Partner coaching was associated with clinically meaningful improvements in participants’ anxiety, and several aspects of HRQOL and health behavior practice during 3 or 12 months [13, 42, 43]. This fact means long-term cancer survivors can benefit from the Health Partner Program [44] at least in relation to distress and HRQOL management within the cancer care continuum. Therefore, these findings leave room for the possibility of improvement of the program and should not discourage development of new programs that allow long-term cancer survivors to be partners with health professionals in the cancer control continuum. Many trials may be needed to learn how to train survivors to be effective health coaches.

Our study has several limitations. First, the completion rate was low (30.7% of the patients did not complete the full 12-month telephone coaching) and this might have negatively influenced primary outcomes [45]. In addition, due to the lack of participants in the 6-month period, we did not include the 6 month follow-up results in the statistical analyses. Second, the participants did not represent the whole cancer population; most of the recruited participants were early-stage (in situ, localized, or regional cancers with a favorable prognosis) cancer survivors and this often leads to “ceiling effects” in which these participants often report little improvement relative to high baselines in a wide range of modifiable health behaviors and QOL items. Third, our measures of diet and PA were based on self-reports and might have therefore included reporting errors. Finally, our participants included a wide range of cancer types, which might have complicated the interpretation of our findings. If our study were done for a single type of cancer, the interpretation of the findings may have been easier. Further studies are needed to develop new creative programs that are more effective.

## Conclusions

Although this program did not change the participants’ primary behaviors such as physical activity or dietary habits, the program was effective in improving cancer patients’ ability to manage their anxiety, social functioning, and symptoms. This health coaching program provides a creative partnership between long term cancer survivors and medical professionals enable cancer patients to manage their distress and QOL with positive growth.

## Additional file

Additional file 1: Table S1. The intervention included 1) a TTM-based health education booklet and work book for cancer survivors, 2) a workshop for empowerment of patients’ leadership skills, and 3) TTM-based telephone coaching with a health coaching manual (repeated assessment of stage of change, and planning how to achieve the health target levels in accordance with their preferences and abilities) are described in the Additional file 1: Table S1. (DOCX 22 kb)

## Abbreviations

CBT: Cognitive Behavior Therapy
HADS: Hospital Anxiety and Depression Scale
HRQOL: Health Related Quality of life
IES-R: Impact of Events Scale-Revised
LEACH: Leadership and Coaching for Health
PA: Physical activity
TTM: Trans-theoretical model
