# Supplementary material for: A randomized controlled trial of physical activity, dietary habit, and distress management with the Leadership and Coaching for Health (LEACH) program for disease-free cancer survivors
Source: BMC Cancer. 2017 May 2;17:298. doi: 10.1186/s12885-017-3290-9 (PMC5412037; doi:10.1186/s12885-017-3290-9)
Supplement: Additional file 1: Table S1. — The intervention included 1) a TTM-based health education booklet and work book for cancer survivors, 2) a workshop for empowerment of patients’ leadership skills, and 3) TTM-based telephone coaching with a health coaching manual (repeated assessment of stage of change, and planning how to achieve the health target levels in accordance with their preferences and abilities) are described in the Additional file 1: Table S1. (DOCX 22 kb) [file 12885_2017_3290_MOESM1_ESM.docx]

Additional file 1: Table S1. Features of Intervention Program

| - ***Health education***: Health education uses the comprehensive, multifaceted core strategies from the trans-theoretical model (TTM) of health behavior change, which assumes that patients develop a new behavior by moving through a series of five stages.[^49^](#_ENREF_49)^,^[^50^](#_ENREF_50) The conceptual TTM framework for intervention contains activities such as consciousness-raising, dramatic relief, self-efficacy building, self-reevaluation, and self-liberation, which help patients for improve in TTM stages. |
| --- |
| - ***Self-leadership***: We modified the “Seven Habits of Highly Effective People”^36^ into the “Seven Habits of Highly Effective People with Cancer” and developed a self-management program around it in the expectation that it would empower cancer patients to proactively manage their disease and accomplish their goals. The seven habits are the following: 1) Be Proactive, 2) Begin with the End in Mind, 3) Put First Things First, 4) Think Win-Win, 5) Seek First to Understand, Then to Be Understood, 6) Synergize, and 7) Sharpen the Saw. Self-leadership is the process by which people influence their own behavior using self-direction and self-motivation.[^51^](#_ENREF_51)^,^[^52^](#_ENREF_52) Therefore, strengthening self-leadership should enable cancer patients to proactively manage their own illness and develop realistic action plans to accomplish their goals. |
| - ***Health coaching***: We defined health coaching as helping patients to gain the knowledge, skills, tools, and confidence to become active participants in managing their own care so that they can reach their self-identified health goals.[^53^](#_ENREF_53) Health coaching focuses on patient's health problem based on the TTM stages of change. It helps patients target their health goal, plan how to achieve the target level in accordance with their preferences and abilities, and identify and deal with obstacles. Coaching skills include contextual listening, discovery questioning, messaging, acknowledging, and celebrating.[^54^](#_ENREF_54) |
